# Permanent Draft Genome Sequence of *Ensifer* sp. Strain LCM 4579, a Salt-Tolerant, Nitrogen-Fixing Bacterium Isolated from Senegalese Soil

**DOI:** 10.1128/genomeA.00117-17

**Published:** 2017-04-06

**Authors:** Nathalie Diagne, Erik Swanson, Céline Pesce, Fatoumata Fall, Fatou Diouf, Niokhor Bakhoum, Dioumacor Fall, Mathieu Ndigue Faye, Rediet Oshone, Stephen Simpson, Krystalynne Morris, W. Kelley Thomas, Lionel Moulin, Diegane Diouf, Louis S. Tisa

**Affiliations:** a Centre National de Recherches Agronomiques, Institut Sénégalais de Recherches Agricoles (CNRA/ISRA), Bambey, Senegal; b Laboratoire Mixte International Adaptation des Plantes et Microorganismes Associés aux Stress Environnementaux (LAPSE), Centre de Recherche de Bel Air, Dakar-Bel Air, Sénégal; c University of New Hampshire, Durham, New Hampshire, USA; d Laboratoire Commun de Microbiologie IRD/ISRA/UCAD, Centre de Recherche de Bel Air, Dakar, Senegal; e Centre National de Recherches Forestières, Institut Sénégalais de Recherches Agricoles (CNRA/ISRA), Hann Dakar, Senegal; f Institut de Recherche Pour le Développement (IRD), UMR IPME 34394, Montpellier, France; g Département de Biologie Végétale, Université Cheikh Anta Diop (UCAD), Dakar, Senegal

## Abstract

The genus *Ensifer* (formerly *Sinorhizobium*) contains many species able to form nitrogen-fixing nodules on plants of the legume family. Here, we report the 6.1-Mb draft genome sequence of *Ensifer* sp. strain LCM 4579, with a G+C content of 62.4% and 5,613 candidate protein-encoding genes.

## GENOME ANNOUNCEMENT

Rhizobia are symbiotic nitrogen-fixing bacteria able to convert N_2_ atmospheric nitrogen into nitrogen compounds in specialized structures on plant roots called nodules (1). In exchange, rhizobia take advantage of carbon substrates derived from plant photosynthesis for their activities (2). The host plants of these nitrogen-fixing bacteria are included in the family *Fabaceae* or *Leguminosae*. The *Leguminosae* family comprises three subfamilies: *Caesalpinioideae*, *Mimosoideae*, and *Papilionoideae* (3–6); each contains genera able to form root nodules. The morphology, habitat, and ecology of legumes are very diverse, ranging from Arctic annuals to tropical trees (7). They are important crop plants that can enrich soil nitrogen and they have protein-rich seeds that are important in human and animal nutrition. In addition to producing valuable food and animal feed, legumes are beneficial as rotational crops, green manure, cover crops, forage, and fuelwood.

Among the rhizobia is the genus *Ensifer* [formerly *Sinorhizobium* (8, 9)], which includes species with high geographical dispersion. These bacteria are able to nodulate a wide variety of legumes. Some *Ensifer* strains are able to develop in soil under different environmental stresses, including salinity (10). One of these bacteria, *Ensifer* sp. strain LCM 4579, was isolated from the rhizosphere of soil around *Prosopis juliflora* under saline conditions (11). Under *in vitro* culture conditions, this strain is able to tolerate up to 600 mM NaCl. The isolate will also form root nodules on *P. juliflora* and *Acacia seyal* plants that actively fix nitrogen. Because of these properties, this strain could be potentially used in association with leguminous plants for the reforestation of saline lands. The *Ensifer* sp. strain LCM 4579 genome was sequenced to provide information on its physiology and ecology, and to identify molecular markers that are involved in its tolerance to salinity.

Sequencing of the draft genome of *Ensifer* sp. strain LCM 4579 was performed at the Hubbard Center for Genome Studies (University of New Hampshire, Durham, NH, USA) using Illumina technology techniques (12). A standard Illumina shotgun library was constructed and sequenced using the Illumina HiSeq2500 platform, which generated 21,371,196 reads (260-bp insert size) totaling 5,129 Mb. The Illumina sequence data were trimmed by Trimmonatic version 0.32 (13) and assembled using Spades version 3.5 (14) and ALLPaths-LG version r52488 (15). The final draft assembly for *Ensifer* sp. strain LCM 4579 consisted of 61 contigs with an *N*_50_ contig size of 247.7 kb and 63.8× coverage of the genome. The final assembled genome contained a total sequence length of 6,137,409 bp with a G+C content of 62.4%.

The assembled *Ensifer* sp. strain LCM 4579 genome was annotated via the NCBI Prokaryotic Genome Annotation Pipeline (PGAP) and resulted in 5,613 candidate protein-encoding genes, 47 tRNAs, and four rRNAs. Annotation of the genome revealed the presence of the *nif* and common *nod* operons involved in nitrogen fixation and host plant nodulation, respectively.

### Accession number(s).

This whole-genome shotgun sequence has been deposited at DDBJ/EMBL/GenBank under the accession number MDDV00000000. The version described in this paper is the first version, MDDV01000000.
